# “Existential Catastrophe Anxiety”: Phenomenology of Fearful Emotions in a Subset of Service Users With Severe Mental Health Conditions

**DOI:** 10.3389/fpsyg.2022.766149

**Published:** 2022-03-10

**Authors:** Didrik Heggdal, Synne Borgejordet, Roar Fosse

**Affiliations:** Division of Mental Health and Addiction, Vestre Viken Hospital Trust, Drammen, Norway

**Keywords:** existential fear, catastrophic feelings, annihilation anxiety, ontological insecurity, affect phobia, exposure therapy

## Abstract

A subset of people with severe mental health conditions feels they are on the verge of losing control, even in the absence of external threats or triggers. Some go to extreme ends to avoid affective arousal and associated expectations of a possible, impending catastrophe. We have learned about such phenomenological, emotional challenges in a group of individuals with severe, composite mental health problems and psychosocial disabilities. These individuals have had long treatment histories in the mental health care system. They have been encountered at a specialized inpatient ward offering exposure-based therapy that aims at restoring self-regulation and recovery. We describe the phenomenology of anxiety and fear presented by these service users, a fear we have coined existential catastrophe anxiety (ECa). We also suggest a set of underlying, interacting, psychological mechanisms that may give rise to ECa, before comparing ECa with three other constructs previously described in the literature—annihilation anxiety, ontological insecurity, and affect phobia. These comparisons show several similarities, but also unique qualities with ECa and its suggested underlying mechanisms. The conceptualization of ECa may aid clinicians in addressing extreme experiential turmoil and engage service users in empowering therapeutic projects.

## Introduction

Some persons with severe mental health conditions struggle with painful feelings of anxiety, fear, alarm, and dread. They may go to extreme ends to avoid and get rid of these feelings, including extensive substance abuse and self-harm. An array of clinicians and researchers have attempted to capture these types of fears using various constructs, models, and theoretical frameworks. Hurvich (2003) used the term annihilation anxieties as a common denominator of dreadful feelings noted by many therapists within the psychodynamic tradition. He defined annihilation anxiety as fear of imminent mental or physical destruction and extinction, which included fear of being overwhelmed, losing control, suffocation, exploding, shrinking, being destroyed, fusion, dissolution, invasion/being overrun, loss of self-cohesion, and feelings of an impending catastrophe. Among this variety of experiences, Hurvich and colleagues considered the most central to be fear of being overwhelmed (Hurvich et al., 1993). Tangential concepts used by other psychodynamic clinicians include traumatic anxiety (Freud, 1926), psychotic anxiety (Klein, 1975), instinctive anxiety (Freud, 1966), schizoid anxiety (Fairbairn, 1952), primary anxiety (Fenichel, 1945; Zetzel, 1949), unthinkable anxiety (Winnicott, 1960), doomsday expectation (Krystal, 1988), nameless dread (Bion, 1962), and disintegration anxiety (Kohut, 1972). To capture a possible overlapping but also different set of experiences, Laing (1965) used the concept ontological insecurity, referring to feelings of being in danger of losing one’s personal connection, self-identity, and autonomy. According to Laing, this would apply when an individual lacks a fixed sense of one’s reality and identity, feeling more illegitimate than real, more dead than alive, completely different, and separated from the rest of the world. By not being able to take their identity for granted, ontologically insecure persons would have a constant fear of their self being destroyed. This would include fear of losing autonomy and become subject to the will of others, a fear of implosion or being wiped out by the “real” outside world, and fear of depersonalization and petrification. In order to preserve oneself as a separate and cohesive person in face of such existential threats, it may feel necessary to isolate from others as one fears that closeness can lead to losing oneself in the Other or in an all-consuming emptiness where one disappears into nothingness (Laing, 1965). Another tangential construct is “affect phobia,” which McCullough et al., (2003; see also McCullough Vaillant, 1997) used to conceptualize fear of distinct affective states, for example, guilt, anger, or sadness. A person with affect phobia expects unwanted feelings to increase and uses avoidance strategies to prevent being overwhelmed.

Over a 20-year period at a closed inpatient ward (open since 2018, World Health Organization, 2021), we have observed that low-functioning service users with severe, composite mental health conditions seem to experience painful, fear oriented feelings, which may be more or less reminiscent of the variously named constructs noted above. The ward provides a novel psychosocial treatment form, Basal exposure therapy (BET), to individuals who have not achieved enduring improvements despite repeated, treatment attempts in ordinary mental health care (Heggdal, 2022). Confronted with these peoples’ apparently intensive, habitual attempts to avoid painful feelings, BET addresses these feelings within the framework of exposure therapy (Heggdal et al., 2016; Hammer et al., 2018). BET explicitly seeks to avoid any use of coercive measures and puts extensive weight on promoting autonomy, self-regulation, and recovery. The Council of Europe, UN, and WHO have highlighted BET as an example of person-centered, rights-based, recovery-oriented approaches in mental health care (Devandas, 2019; Council of Europe, 2021; World Health Organization, 2021).

We aimed, first, to describe the pervasive or basic fear encountered in persons treated in BET, a fear we have coined existential catastrophe anxiety (ECa). Our second aim was to draw on therapeutic experiences with these individuals to suggest developmental origins of ECa. Third, we aimed to evaluate whether ECa demarcates a unique subjective landscape or condition, compared to three constructs that we have found to lie the closest to ECa – annihilation anxiety, ontological insecurity, and affect phobia.

## Capturing the Phenomenology of Existential Catastrophe Anxiety

We offer BET to persons with severe and composite mental health problems who have long histories of prior treatment attempts in mental health care (Heggdal et al., 2016; Hammer et al., 2018; Heggdal, 2022). Their treatment histories typically include numerous or lengthy inpatient admissions, long-term use of psychotropic drugs, polypharmacy, and extensive use of coercion, with only limited, enduring positive effects (Hammer et al., 2018). In terms of common diagnostic labeling, these individuals present with various disorders, including schizophrenia spectrum disorders, dissociative disorders, bipolar disorders, and complex post-traumatic stress disorder (PTSD), usually with one or more comorbid diagnosis, for example, personality disorders, substance abuse and eating disorders (Heggdal et al., 2016). Characteristic aspects of their clinical picture are delusions, hallucinations, dissociation, and mood fluctuations, typically in combination with (repeated) deliberate self-harm and suicide attempts (Heggdal et al., 2016; Hammer et al., 2018).

BET is a psychosocially oriented inpatient treatment. The aim is to help people who suffer from severe mental health challenges to replace avoidance strategies with acceptance as their primary coping strategy. Thus, BET represents an alternative to the more typical medication-oriented approaches that our service users previously have encountered. The goal in BET is to strengthen patients’ autonomy and self-regulation, with a central focus on the key role of service users in their own improvement. Most service users are voluntarily admitted to the ward, with a few exceptions of individuals who are involuntary admitted to acute wards before transferred to BET.

In BET, we make a clear distinction between exploration of inner phobias and the subsequent trials of exposure. While exploration is going on at a cognitive level, we use behavioral experiments in exposure to invite service users to allow for affective arousal, that is, to experience the bodily emotional sensations associated with inner phobias. It should also be noted that use of force in exposure therapy may result in re-traumatization, and any form of coercion or persuasion may undermine the possibility of therapeutically successful exposure to inner phobias. Therefore, in BET, exposure always is the consequence of a deliberate choice made by the service user and based on his or her values and wishes for a better life. A further prerequisite for exposure is a solid therapeutic relationship. The relational context has the function of a secure base (Bowlby, 1988) with room for any sensations and emotional experiences that transcend service users own coping capacity.

When we have engaged service users in BET in therapeutic explorations, it has become evident that they often experience a pervasive fear, existential threat, or terror, but which they struggle to describe in precise terms. Closer investigation reveals overwhelming fears of dissolving, disintegrating, or falling apart, intrusive beliefs that something terrible is going to happen, such as being destroyed, wiped out, or exploding, and feelings of being engulfed in all-encompassing emptiness or stuck in eternal pain. These individuals seem to have attempted, for a long time, to avoid affective arousal, which gradually has blurred their inner experience and left them with an unclear, distant feeling of impending catastrophe. Below are statements from former users of BET that illustrate this experience, provided as contributions to a recent book and to a qualitative study of their experiences with BET.

“*It did not take many days after a prior admission before the emotional chaos and panic of not having control became too great. I did not dare to let go of the unpleasant feelings - they had to be suppressed at all costs. If I could not calm the chaos, I was sure that I would not be able to live any longer, the discomfort was so strong. The only way I could tame the discomfort was to take everything I had of pills, which in turn led to a new dramatic admission to the emergency ward for pumping, and then a new short-term admission to mental health care*” (Hammer et al., 2020, p. 74).

“*Letting myself sink voluntarily into my deepest abysses did something to me. I thought I was going to cease to exist*” (Hammer et al., 2020, p. 96).

“*In therapy groups, I could get soaked by sweat in my struggle to hold back tears. I was sure that if I started crying, I would never stop, and that I would lose all control. Deep down in the therapeutic process, it was unclear to me what this fear was really about*” (Hammer et al., 2020, p. 81).

“*It was my feelings that I was afraid of. Because I could not cope with my feelings, I started to self-harm and have eating problems. Then all this became stronger, and it got more dramatic. It is like with drugs, when you need more to be able to live and feel alive and also to keep away the feelings that you are so afraid of*” (Heggdal et al., 2015, p. 124).

While such single and anecdotic statements indicate how ECa is experienced at an individual level, the delineation and delimitation of the phenomenon as a psychological construct required that we started out with a broader and more differentiated information base. The clinical working hypothesis in BET is that service users’ excessive, long-term efforts to avoid an impending existential catastrophe have made the phenomenological content of the associated feelings distant and blurred, and at the same time increased fear intensity. Moreover, service users often first convey their phenomenological landscapes of ECa in later phases of treatment, when working alliance is secured and users and therapists engage in working toward common goals of delineating emotional experiences that can be targeted with exposure therapy. Thus, this article draws on condensed information from explicit clinical explorative work with service users at the 24/7 BET ward.

First, we invited experienced therapists and staff at the BET section to provide examples of how the many service users they had worked with over the years described their inner life. The examples should include various expressions of fearful emotional experiences, as well as service users’ difficulties in grasping or describing their innermost fears, and their motivations and attempts to control and avoid sensations, thoughts, and feelings. The phenomenological approach used in BET aims at description without preconception or prejudice, placing focus on the subjective phenomena while as far as possible reducing intrusive suppositions and speculation that lie outside direct experience (The Lancet, 2021). In exploring inner phobias, BET therapists follow a strict procedure not to destabilize the individual or induce imageries and expectations that are not genuinely her own. The typical sequence is as follows: Therapists invite the service user to talk about a recent event where she experienced distress and used self-destructive coping strategies. The therapist depicts the here-and-now context and invites her to look back, from a distant perspective, and together explore what happened. The service user describes how she experienced the situation and what she did at a behavioral level. Next, the therapist typically asks, “what would have happened if you in that specific situation had chosen *not to* engage in avoidant behavior, for example, self-harm?” When repeating this procedure, in the end, service users will describe what they imagine might happen if refraining from all kinds of avoidant behavior, which will reveal the individual phenomenology of the inner phobia. We complete the exploration by assessing to what degree the individual, at an intellectual level, actually believes the catastrophe will happen. The individual’s degree of cognitive fusion with her expectation is decisive for defining the next steps in the therapeutic process, in line with clinical principles in Acceptance and commitment therapy (ACT; Hayes et al., 1999).

We assembled experienced therapists and staff at the BET ward to three rounds of collective brainstorming to derive at examples of statements of service users’ existential catastrophic fear. The brainstorming resulted in a list of several hundred verbal descriptions of how catastrophic fear may be experienced. Following this, we assembled, compared, sorted, and integrated the descriptions of catastrophic fear, before presenting them to two persons with lived experience who recently had completed BET. These two former service users ranked all expressions in terms of their relevance in capturing catastrophic fear. Table 1 shows the 40 expressions they judged to be most relevant.

**Table 1:** Expressions that reflect catastrophic feelings or maneuvers to avoid them.

Feelings just float around inside me, and it is difficult to really know what I feel
I wish my body had no needs
Even when someone attempts to reach me, it is as if I am unavailable deep inside
Intense feelings to me are mostly about fear
Feelings usually come suddenly
If I experience my feelings as threatening, I have to rapidly do something before it ends in chaos
I am afraid to lose control
I have given up my attempts to reach other people
If I allow for what is within me to appear, I will be engulfed by emptiness
Feelings quickly become much too intense
I would have felt less alone if other people really had understood how dramatic my inner life is
If I stay conscious to the pain, I can get stuck in it, and it will never pass
I know I have a body, but it is as if it exists independent of me
I think it is best to feel as little as possible
It is best to keep occupied with something so that I do not start thinking on how things really are
My feelings are like a big chunk of all kinds of feelings which are difficult to sort apart
My body and my experiences must be kept apart for me to survive
I try to avoid as best I can that my feelings lead to something catastrophic
I often have a feeling of extreme danger that comes without warning
I am afraid of what can happen if I let go of my control
I think it is better to be numb than to have to feel everything all the time
I am engulfed by emptiness
I fear that if I let go of control over my feelings, I will dissolve and become nothing
The pain inside me will fill me completely and take up all space, if I stop fighting it
To me, a good day is a day completely without feelings
Good feelings can suddenly change into negative feelings without me understanding what is happening
It is as if I live within a thick shell, without feeling protected by it
It is best to be occupied with something so that chaos does not take the upper hand
If I really should allow my inner fear to rise to the surface, I would break into pieces and never become myself again
I always need to have something at hand, so that I can stop my feelings before they take over completely
My body feels strange and foreign
I have lost hope, nothing matters
Nobody sees me
It is difficult to get into real contact with other people
I feel I am stuck, as in a bubble of jelly
Intense feelings to me are mostly about bodily discomfort
I will become mad if I am to allow my feelings to ravage freely
I am afraid to disappear if I loosen up on my control
I make sure to avoid things and situations that I know make me stressed

One may view the phenomenology of ECa and the service users’ relation to these feelings in terms of both an emotional and a cognitive component. The emotional component of ECa appears as an incomprehensible, fluctuating, and undifferentiated affective state experienced as anxiety, psychological pain, and a feeling of being under existential threat. It includes the sensation that the condition will escalate and get worse, as well as an urge to prevent further increase in affective arousal. The cognitive component is a motivation and ideation to prevent affective arousal and the experience of existential threats. Furthermore, the cognitive component includes the belief that failure to do this in its extreme consequence may lead to existential catastrophe. In this sense, we consider the feared existential catastrophe as a phobic object.

## Hypothesis of Developmental Mechanisms

**Figure 1:**
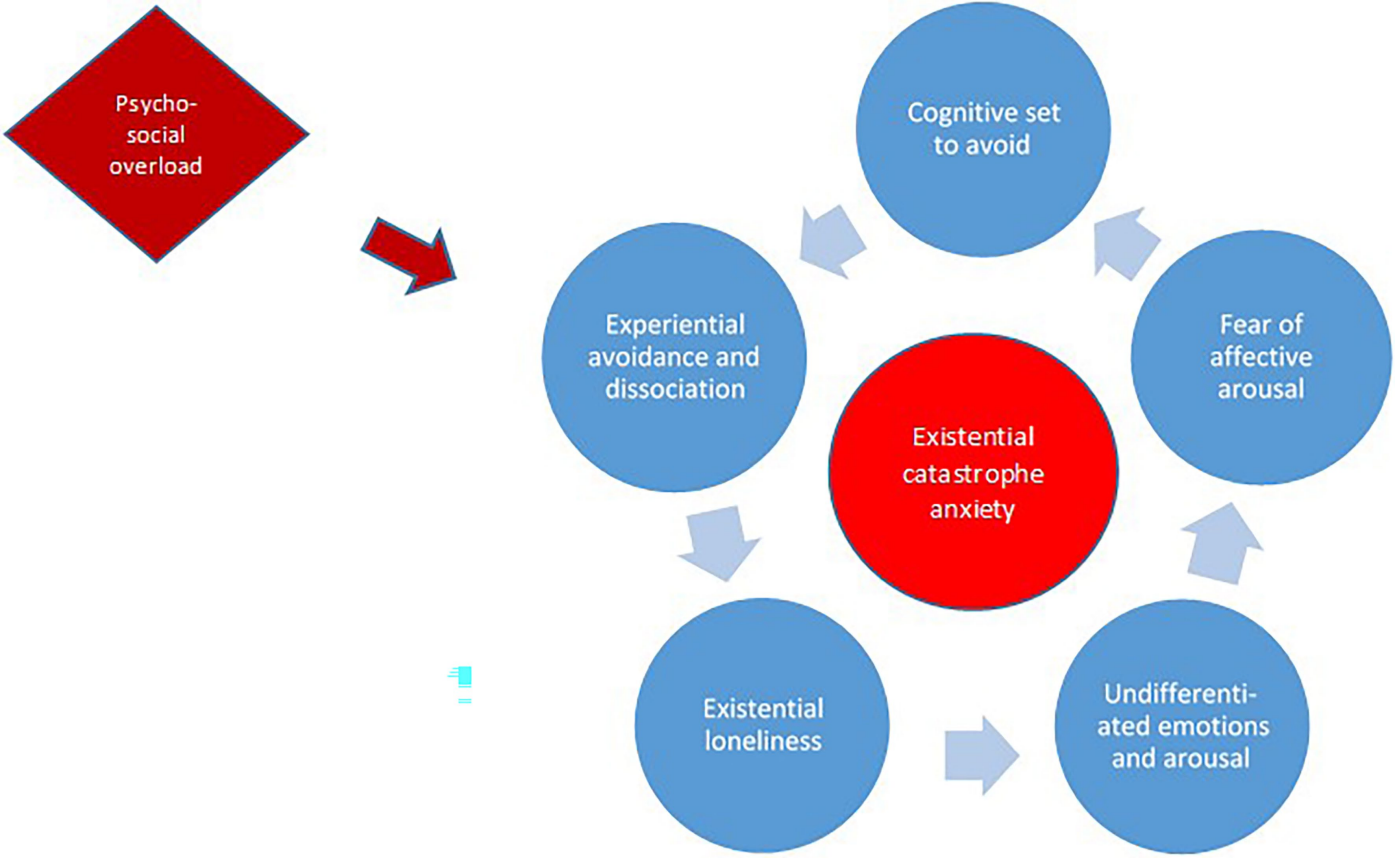
Hypothesized interacting processes in developing and maintaining existential catastrophe anxiety. See main text for descriptions.

In conceptualizing developmental origins of ECa, we draw on more than 20 years of clinical experience with over 150 service users in BET who suffered from severe mental health challenges. Statistically, severe mental health challenges are associated with relational trauma (van der Kolk et al., 2005; Briere and Scott, 2015). Anamnestic data confirm high incidences of abuse and neglect experiences in the histories of most BET service users. In the clinic, these people convey a profound experience of loneliness. Some say they have been left alone with their traumatic memories because of mental health care’s one-sided emphasis on symptoms. Others explain that they withdraw from others to protect themselves from being subjected to further traumatizing events. We assumed that experiences of complex relational trauma or severe psychological overwhelm, combined with insufficient psychosocial protection and support, are original causes which set the scene for later development of ECa. We hypothesize that a dynamic relationship between a set of temporally proximate, closely intertwined psychological processes creates, maintains, and amplifies inner phobias, with existential catastrophe anxiety as an extreme end product. Below, we describe five components that are consistent with and informed by both the phenomenology of ECa, the service users’ associated maneuvers, and a complex trauma perspective. The five components and our hypothesis about their typical pattern of interaction are depicted in Figure 1.

### Experiential Avoidance and Dissociation

In the absence of adequate protection and support (a secure base), when traumatic experiences take place, individuals must cope with emotional consequences on their own. In this context, psychosocially challenging situations that instigate emotional overwhelm can make it insufficient with both normal coping strategies and neurotically conditioned coping strategies, such as repression and displacement. As a result, the overwhelmed person may distance herself from the experiences using dissociative responses, such as disconnection. This includes shut-off of normal parts of experience that usually are taken for granted, and altered memory, attention, and identity experiences (Holmes et al., 2005; Kihlstrom, 2005). Due to the overwhelming nature of the experiences, the lack of a secure base, and use of dissociation as a way of coping, conditional depersonalization and derealization may occur. This includes feeling detached from one’s sense of self, body, thoughts, and emotions and feeling detached and alienated from one’s environment, as well as experiencing the external world as unreal, emotional numbness, and lack of ownership to personal information. Together, this can come forward as a feeling of not being alive, as if the body is an empty shell, but with the insight that this has to do with subjective experience rather than objective reality (Hunter et al., 2003; Phillips and Sierra, 2003; Sierra and David, 2011).

### Existential Loneliness

A person who feels alienated both from herself and the world around her may experience a being in the world characterized by existential loneliness (Laing, 1965; Bolmsjo et al., 2019). Existential loneliness is a way of being or position in the world where one feels both excluded and actively chooses to exclude oneself as a protection against becoming emotionally overwhelmed. It may appear that one engages in social situations, but for some reason ends up on the outside of social life rather than being genuinely emotionally involved (Laing, 1965; Bolmsjo et al., 2019). In the existentially lonely condition, one excludes oneself from continuous interactions where people mirror and acknowledge each other’s emotional experiences.

### Undifferentiated Emotions and Arousal

A person exposed to overwhelming experiences, and who is existentially lonely, will necessarily react emotionally. However, lack of feedback and affirmation that promote affect awareness, can contribute to making it more difficult to distinguish between emotional qualities, such as frustration vs. longing vs. sadness (Monsen et al., 1996; Barrett et al., 2001; Kang and Shaver, 2004; Mohaupt et al., 2006). The person may experience emotional reactions and qualities as more and less psychophysiological discomfort, as inner pain without any further content, rather than as emotions acknowledged consciously as distinct feelings. Reduced awareness of emotions makes them and their origins less understandable and thus more difficult to regulate (Spitzer et al., 2006). In addition, feelings that are recognized in an affect conscious sense can be associated with or infiltrated into other emotions, for example, joy and engagement may trigger feelings of guilt, shame, or anxiety (McCullough et al., 2003). This may lead the total emotional experience to become undifferentiated, conflicted, or “cloudy.”

### Fear of Affective Arousal

We assume that when individuals with mental health conditions experience emotions as contradictory, painful, and uncontrollable, this may lead to fear of feeling in itself. In such cases, emotional experiences may not help the individual to understand herself or navigate in a complex world. Rather, one may experience that “if I feel less, it will be less bad.” The psychophysiological intensity of emotional experiences might become the primary or only parameter used to evaluate emotional experiences as positive or negative. When affective arousal becomes uncomfortable or frightening, or non-acceptable (Merwin et al., 2010), regardless of emotional qualities, the person will begin to protect herself from feeling anything at all.

### Cognitive Set to Avoid

At a cognitive level, habitual fear of affective arousal may make the person inclined to avoid an increase in arousal at all costs in order to avert potentially overwhelming psychophysiological distress. Such a cognitive mindset where one orients oneself toward avoidance, interrupts, and limits the person’s ability to achieve corrective experiences when dealing with inner discomfort or pain (Foa and Kozak, 1986).

Our impression is that the five component processes may interact in various and individual ways. However, based on our clinical observations we suggest that experiential avoidance and the alienation process that follows constitute basic components in the development of ECa. Furthermore, depersonalization and derealization may lead to existential loneliness, which may contribute to a low degree of affect awareness (Monsen et al., 1996). Next, undifferentiated emotions might fuel a fear of affective arousal, which over time results in an inclination to avoid. This inclination will maintain the experience of alienation, and the circle is completed (Figure 1).

Because avoidance behavior in the short term leads to some sort of relief or keeps the affective arousal in check, the individual’s behavioral response over time will enter a positive feedback loop (Bateson, 1972). This may result in ECa, which further reinforces the pathology-sustaining dynamics between the above-noted components. While we hypothesize that the components influence each other in the order depicted above and in Figure 1, this is a matter of abstracting and simplifying very complex psychological processes. There are possibly several “ways in” to ECa, and dynamics between components might be bi-directional or change over time.

## Discussion

Below, we compare ECa with three existing constructs—“annihilation anxiety,” “ontological insecurity,” and “affect phobia.” We examine relationships between constructs in terms of phenomenological specifics and explanatory, developmental models.

### Annihilation Anxiety

Hurvich and colleagues defined annihilation anxiety as fear of imminent mental or physical destruction. In their definition, they included feelings of being overwhelmed, invaded or run over, fusing with others, losing control and loss of coping abilities, loss of needed support, suffocating, exploding, dissolving, loss of self-cohesion, and fading away, and feelings of an imminent catastrophe (Hurvich et al., 1993; Hurvich, 2003). A varied literature on annihilation anxiety emphasizes different elements, but three dimensions are commonly noted. First, a mental content, imagination, or cognitively oriented expectation of an event that will threaten self-integrity and survival. Second, affect intensity and associated feelings of disorganization, diffuse feelings of helplessness, and overwhelm. Third, susceptibility to traumatic conditions, with a distinction between traumatic arousal and the expectation that a traumatic condition will occur. Hurvich and colleagues have emphasized that annihilation anxiety typically is relevant to psychosis, but also that it plays a role in a wider spectrum of psychopathology, generating and fostering untreatable resistance, rigid character styles, negative self-images, and maladaptive interpersonal patterns (Hurvich et al., 1993; Benveniste et al., 1998). They also asserted that when persons with mental health conditions attempt to neutralize annihilation anxiety, ensuing feelings of hopelessness and uselessness might lead to panic, loss of functions, aggressiveness or avoidance behavior, and self-harm or self-destructive behavior.

Hurvich and colleagues held the affective dimension of annihilation anxiety to include a variety of intense feelings of dread, fear, and anxiety, in addition to diffuse feelings of being overwhelmed. This may encompass the incomprehensible, fluctuating, and undifferentiated state of emotion experienced as anxiety, pain, and existential threat in ECa. In addition, common to the two phenomena is the urge to dampen or keep affective arousal in check. Moreover, what is feared appears in essence to be similar in the two constructs—imminent mental or physical destruction, existential catastrophe, annihilation, and an array of the most prominent experiential correlates might be compatible as well. However, the experience we refer to as ECa may be more homogenous than annihilation anxiety in terms of both clinical expression and developmental course. Moreover, the cognitive image of imminent catastrophe in ECa appears not to be mirrored by equally specified images of what annihilation anxiety is associated with. Still, one might understand annihilation anxiety as an umbrella term, covering a wider spectrum of experiential states that among others includes the phenomenology of ECa.

According to Hurvich and colleagues, the nature and course of annihilation anxiety may vary, at times arising “out of the blue,” as a sudden fear reaction not preceded by any cognitive expectation of annihilation. In other instances, its nature and course may appear as enduring expectations of annihilation and feelings of anxiety (Hurvich et al., 1993; Hurvich, 2000). Based on our own clinical work, persons treated with BET may not continuously expect a catastrophe to be imminent. What rather may be continuously present to our users is the tendency to avoid affective arousal. When we more closely explore emotions and avoidance behaviors, an associated cognitive component of expectation may become evident. At times, the level of anxiety experienced by our service users appears to increase suddenly, but at the same time to be present at moderate intensity in an enduring, basic way. In this sense, ECa may be compatible with both forms of nature or course that characterize annihilation anxiety. A possible difference might be that inner turmoil rarely is completely absent in users of BET.

In terms of underlying mechanisms, Hurvich and other psychoanalytic writers have portrayed annihilation anxiety as rooted in early traumatic experiences, in line with what we do for ECa. Consistent with this portrait, Schiek-Gamble and Hurvich (2015) found higher levels of fear of extinction in people who had experienced child abuse and accumulated negative life events, such as early loss of a parent, parental conflict, and bullying. Also held to contribute to annihilation anxiety, consistent with general psychoanalytic theory, is ego weakness, understood as reduced capability to control impulses and tolerate frustration, disappointment, and stress (Lake, 1985). Individuals with ego weakness easily would experience anxiety and conflicts, have an excessive or immature use of defense mechanisms, and be inclined to develop neurotic symptoms (Hurvich et al., 1993; Hurvich, 2000). Furthermore, psychoanalytic writers associate annihilation anxiety with object loss (of people one is emotionally attached to), and with a disturbed sense of self (self-pathology; Benveniste et al., 1998). While likely including overlapping aspects, these underlying mechanisms differ somewhat from those we have outlined for ECa.

### Ontological Insecurity

Laing (1965) introduced the concept ontological insecurity as a potentially universal feature of the human condition that characterizes vulnerability to psychosis. Ontologically insecure persons experience feelings of danger of losing their personal connection, self-coherence, self-identity, and autonomy. They will not feel able to take their identity for granted, and experience themselves as more unreal than real, more dead than alive, different, and separated from the rest of the world. The borders between the self and others are unclear, thus a source of fear of destruction. Laing highlighted three implicated fears: (1) That oneself, one’s autonomy, will be swallowed up, leaving one as subject to the will of others, (2) fear of implosion, of being wiped out by the “real” outside world, and (3) fear of petrification and depersonalization. For self-preservation, the ontologically insecure person might try to divide herself in two by withdrawing from the body into an inner, private fortress of the mind, hoping to be safe from threats of extinction, being swallowed, implosion, petrification, or depersonalization (Cooper, 2003). This would leave the person with a dichotomized, depersonalized body, and a false self, a decoy, in the face of others, a shell but at the same time a way of being with others that leaves the real self in peace. In this perspective, as the true self withdraws further and further, the individual may cross the line into psychosis. Here, self-harm can appear as an attempt to overcome feelings of physical numbness and fear of self-destruction. The notion of strong associations between ontological insecurity and psychosis is supported empirically (Marlowe et al., 2020).

According to Laing, antecedents of ontological insecurity are experiences of existential loneliness early in life. Loneliness would get in the way of developing a basic self-perception and self-autonomy, in turn leading to perceiving the self as threatened. Among vulnerability factors, Laing included having been in a care situation where one was ignored, being met with a neutral attitude, inability to influence those around, and inconsistency in feedback from others about what they expect and who one is as a person. These features partially overlap with the psychosocially adverse experiences we hold as original causes to ECa. Also thought to lead to ontological insecurity is symbiotic functioning with the caregiver, not due to lack of mirroring but to unclear self-delimitation in the child–parent dyad (Laing, 1965).

A core similarity between ontological insecurity and ECa is the centering on existential threat, with the fear of existential catastrophe seen as present also in the ontologically insecure person as fear of immersion, implosion, petrification, or complete depersonalization. Another, apparent phenomenological similarity are feelings of numbness and doubt as to whether one exists at all. In service users in BET, this is often part of what feels threatening, and it may lead to self-harming behavior in an attempt to achieve self-anchoring. Further characterizing ontological insecurity are perceptions that other people and the outside world are dangerous. This is reminiscent of our inclusion in ECa that encounters with others may trigger threatening affective arousal.

In terms of underlying mechanisms, existential loneliness holds a central position in both ECa and ontological insecurity. However, the two constructs highlight different inner experiences as triggers of feelings of existential threat. In ECa, the driving force behind states of anxiety is the fear of affective overwhelm that leads to existential catastrophe. In ontological insecurity, the experience of alienation seems to be what triggers fears of self-destruction. Moreover, while depersonalization and derealization operate in both states and constructs, in ECa these components contribute to development of existential catastrophe anxiety. When it comes to ontological insecurity, depersonalization and derealization represent the main reasons for perceiving the self as threatened.

Important to ontological insecurity is the unclear self-delimitation in encounters with others, with the individual oscillating between distance and closeness in attempts to protect oneself against extinction. This leaves, as two pitfalls, destruction due to either total proximity or total isolation. We have not included this dynamics in ECa. However, our service users may end up in interactions with healthcare professionals that is possible to understand in this perspective.

### Affect Phobia

Affect phobia is a term often used in short-term dynamic psychotherapy, developed as an alternative to the classic psychodynamic term “conflicts” (McCullough et al., 2003). McCullough defined this concept as a phobia of affects that produce anxiety, with underlying mechanisms rooted in the activating and inhibiting functions of emotions. Activating emotional effects energize and facilitate action, for example approaching rather than avoiding, opening rather than closing, and running rather than stiffening. Inhibitory effects of emotions make us stop, withdraw, and tighten rather than let go.

McCullough et al. (2003) suggested that affect phobias develop because excessive constraints are placed on a child’s adaptive activating affects, such as anger, joy, and excitement. The child may learn that in order to be loved or escape punishment, these emotions are not allowed, which conditions them to inhibitory affects, such as shame and guilt. This may leave the person with fearing and trying to suppress and evade activating emotional reactions, resulting in affect phobia and excessively inhibited behavior. Consequences include difficulties in setting boundaries and self-defeating behavior in relationships. Violent outbursts of anger can appear when suppressed feelings come to the surface. Pathologic functioning may also occur in the absence of inhibitory emotions to regulate action and reaction patterns, as seen with excessive impulsive behavior (McCullough Vaillant, 1997). Essential in the treatment of affect phobia is guiding the individual toward adaptive affects and affect expression on both the intrapsychic and interpersonal level, using affect exposure as a basic intervention (McCullough et al., 2003).

A central similarity between affect phobia and ECa is the internal phobia perspective, including fear of affective arousal and associated avoidance maneuvers. Therapeutically, in both McCulloughs’ treatment model and in BET, this indicates exposure therapy as the proper treatment form. What may differentiate affect phobia from the phenomenal landscape we coin ECa, is the nature of the phobic states. The phobic object in affect phobia is the dreaded states of emotions, and fear has to do with distinct emotional states, such as anger, joy, and excitement. In ECa, threat is understood as feelings and expectations of an impending existential catastrophe; specifically, that undifferentiated affective arousal and emotional overwhelm, if not avoided, will lead to existential catastrophe. Here, we would not link fear to distinct feelings, and we characterize the emotional component as undifferentiated affective arousal. In addition, we have observed a persistent experience of anxiety in people who struggle with ECa, linked to expecting that arousal may increase, followed by an urge to stagnate such an increase. In contrast, affect phobia may not always involve a subjective experience of anxiety but can instead be expressed as, for example, shame. However, both conditions may include the expectation that unwanted affective arousal would increase if it were not prevented.

During treatment courses in BET, we have observed that service users increasingly become conscious of affects and recognize distinct feelings that they fear. This may support the description of their prior condition of ECa as an extreme and far-reaching affect phobia, where the phobic object as well as subjective experience over time had become vague and unclear. Here, we speculate that the ability to experience feelings as distinct initially was present in our service users, but that attempts to avoid arousal gradually made feelings more diffuse. Thus, one might think of affect phobia and ECa as representing different phases or degrees of maladaptation on a psychopathological continuum. When individuals improve during clinical work in BET, they could be understood as moving from severe to less severe positions on such a continuum. The cause of such movements of improvement could be experiences throughout the therapeutic process with turning attention inwards, over time leading to an increased affect awareness. This might lead to the appearance of an ability to recognize fear of distinct emotions—which is the core of affect phobia—as an intermediate step toward more complete recovery. That said, we do not know whether service users in BET, when improving, are *re*gaining previously acquired but subsequently lost emotional competence, or, alternatively, for the first time reach emotional achievements they never had acquired earlier in life.

## Concluding Comments

Through clinical practice with BET, we have noted an extremely fearful, phenomenological landscape in a group of low-functioning service users with composite, severe mental health conditions, who typically have not improved in spite of repeated, prior treatment attempts. We have coined these fearful experiences as existential catastrophe anxiety, which we assume originate from traumatizing psychosocial overload during childhood and adolescence. Subsequent to trauma, we hypothesize that five interacting psychological processes or factors lead up to the emergence of ECa: experiential avoidance and dissociation, existential loneliness, feelings that become experienced as undifferentiated bodily arousal, fear of affective arousal, and a cognitive set or preparedness to avoid.

ECa largely appears to be covered by the construct annihilation anxiety, which, at the same time, seems to capture a substantially broader phenomenon than ECa. In describing underlying mechanisms of annihilation anxiety, Hurvich and colleagues referred to common psychoanalytic factors and processes that seem to differ somewhat from the mechanisms we have suggested for ECa. However, one needs a more in-depth analysis of conceptual usage and developmental dynamics to determine the degree of overlap between mechanisms of annihilation anxiety and ECa. Moreover, ECa appears to contain the experience of existential threat as depicted in Laing’s notion of ontological insecurity. Furthermore, ECa shares several features with McCullough’s concept affect phobia (McCullough et al., 2003). The latter includes both an internal phobia perspective, fear of affective arousal, and avoidance maneuvers. However, while affect phobia is associated with distinct feelings, the phobic object we have labeled ECa is characterized by undifferentiated psychophysiological emotional experiences.

The phenomenology and explanatory factors we have suggested for ECa may delineate basic features of some peoples’ extreme experiences in a manner that is clinically useful.

The suggested underlying processes may provide possible guiding principles and “entrances” for therapeutic interventions to address pathology-maintaining dynamics in individuals with severe mental health conditions and psychosocial disabilities who otherwise may be considered impossible to treat. Thus, the model and its underlying components may bring forward or visualize different clinical paths for working with these service users. However, the ECa model is currently a theoretical construction that needs further empirical investigation and validation.

We have described the dynamic between five components of ECa as a marginalizing vicious circle, and ECa as the extreme end product of psychosocial maladaptation over time. Although the suggested circular relationship between the components may be too simplistic, our hypothesis and presentation of ECa may inspire further investigation that can help clarifying causes and mechanisms associated with fearful inner experiences.

## Data Availability Statement

The original contributions presented in the study are included in the article/supplementary material, further inquiries can be directed to the corresponding author.

## Author Contributions

DH has developed the BET model and the hypothesized developmental pathways leading to existential catastrophic anxiety. SB has led the comparison of existential catastrophe anxiety with other clinical constructs. RF contributed to phenomenological descriptions of the construct and developed complete drafts and led revisions of the manuscript. DH and SB provided text fragments and paragraphs to be included in various drafts. All authors contributed to the article and approved the submitted version.

## Conflict of Interest

The authors declare that the research was conducted in the absence of any commercial or financial relationships that could be construed as a potential conflict of interest.

## Publisher’s Note

All claims expressed in this article are solely those of the authors and do not necessarily represent those of their affiliated organizations, or those of the publisher, the editors and the reviewers. Any product that may be evaluated in this article, or claim that may be made by its manufacturer, is not guaranteed or endorsed by the publisher.
